# What Works Where and How for Uptake and Impact of Artificial Intelligence in Pathology: Review of Theories for a Realist Evaluation

**DOI:** 10.2196/38039

**Published:** 2023-04-24

**Authors:** Henry King, Judy Wright, Darren Treanor, Bethany Williams, Rebecca Randell

**Affiliations:** 1 Faculty of Medicine & Health University of Leeds Leeds United Kingdom; 2 Leeds Teaching Hospitals NHS Trust Leeds United Kingdom; 3 Department of Clinical Pathology, and Department of Clinical and Experimental Medicine Linköping University Linköping Sweden; 4 Center for Medical Image Science and Visualization Linköping University Linköping Sweden; 5 Faculty of Health Studies University of Bradford Bradford United Kingdom; 6 Wolfson Centre for Applied Health Research Bradford United Kingdom

**Keywords:** artificial intelligence, AI, machine learning, histopathology, pathology, implementation, review

## Abstract

**Background:**

There is increasing interest in the use of artificial intelligence (AI) in pathology to increase accuracy and efficiency. To date, studies of clinicians’ perceptions of AI have found only moderate acceptability, suggesting the need for further research regarding how to integrate it into clinical practice.

**Objective:**

The aim of the study was to determine contextual factors that may support or constrain the uptake of AI in pathology.

**Methods:**

To go beyond a simple listing of barriers and facilitators, we drew on the approach of realist evaluation and undertook a review of the literature to elicit stakeholders’ theories of how, for whom, and in what circumstances AI can provide benefit in pathology. Searches were designed by an information specialist and peer-reviewed by a second information specialist. Searches were run on the arXiv.org repository, MEDLINE, and the Health Management Information Consortium, with additional searches undertaken on a range of websites to identify gray literature. In line with a realist approach, we also made use of relevant theory. Included documents were indexed in NVivo 12, using codes to capture different contexts, mechanisms, and outcomes that could affect the introduction of AI in pathology. Coded data were used to produce narrative summaries of each of the identified contexts, mechanisms, and outcomes, which were then translated into theories in the form of context-mechanism-outcome configurations.

**Results:**

A total of 101 relevant documents were identified. Our analysis indicates that the benefits that can be achieved will vary according to the size and nature of the pathology department’s workload and the extent to which pathologists work collaboratively; the major perceived benefit for specialist centers is in reducing workload. For uptake of AI, pathologists’ trust is essential. Existing theories suggest that if pathologists are able to “make sense” of AI, engage in the adoption process, receive support in adapting their work processes, and can identify potential benefits to its introduction, it is more likely to be accepted.

**Conclusions:**

For uptake of AI in pathology, for all but the most simple quantitative tasks, measures will be required that either increase confidence in the system or provide users with an understanding of the performance of the system. For specialist centers, efforts should focus on reducing workload rather than increasing accuracy. Designers also need to give careful thought to usability and how AI is integrated into pathologists’ workflow.

## Introduction

Pathologists diagnose cancer and other diseases by using a microscope to examine glass slides containing thin sections of human tissue. They perform a variety of tasks including recognizing patterns at high and low power, counting and measuring particular features, and using this information to classify, grade, and stage tumors. Until recently, pathology had remained largely unchanged since the specialty first emerged over 100 years ago [1,2]. While there had been huge advances in how tissue is stained and visualized, with the discovery of new chemicals and the development of labeled antibodies, the fundamental process of morphological assessment of tissue had not altered, with the microscope remaining as essential as it ever was [1]. However, technological advances over the past 20 years mean that it is now possible to scan slides quickly and at high resolution so that they can be viewed on a computer display through the use of whole slide imaging (WSI) [3,4]. This echoes the digitization of radiology but with one considerable difference; WSI necessitates an analog (glass slide) to digital (WSI image) conversion, whereas radiology captures images in digital format [5]. These digital images can be subjected to computer-based image analysis, which can aid pathologists in a range of areas such as biomarker quantification, object measurement, and object counting (cells or nuclei) [6,7].

There has been widespread interest in developing artificial intelligence (AI) image analysis tools in pathology [8]. The combination of WSI and AI means there is not only the possibility of replacing the microscope but also radically altering the role of pathologists. However, while there are a number of commentaries about the challenges of introducing AI into health care [9-11], there are few empirical studies of the implementation of AI into practice [12-14]. A recent scoping review of studies of perceptions of AI among clinicians, patients, and the public, which identified 26 studies, found moderate acceptability, but a number of concerns were identified, including a lack of trust in patient safety and technology maturity [14]. Only 11 of the 26 studies included health care staff, and none explored the perceptions of pathologists. This review explores the current literature in order to understand stakeholders’ perspectives on factors that may support or constrain the implementation and uptake of AI in pathology. Given the lack of literature on pathologists’ perceptions of AI, we have drawn on the approach of realist evaluation, allowing us to make use of a wider range of literature, including relevant theory.

## Methods

### Study Design

The use of AI in health care can be characterized as a complex intervention comprising a number of different elements that act both independently and interdependently. These include technological (eg, functionality and user interface), organizational (eg, implementation process, including training and support), and social components (eg, staff attitudes). Studying complex interventions requires a strong theoretical foundation [15]. Realist evaluation is a theory-driven approach to understanding for whom and in what circumstances complex interventions work [16] and has been used for studying a number of complex interventions, including health information technology [17]. Realist approaches can be used both to evaluate complex interventions and inform intervention design [18]. For these reasons, a realist approach is highly appropriate for the conduct of this review.

Technology depends on human agency to work; technology in and of itself does not cause change, it is how people choose to make use of (or not) the resources that a technology offers to them that lead to what we typically consider to be the impacts of technology. Such choices are highly dependent on context. So, while a technology may provide the desired impact in one context, it is unlikely to produce the same impact across all settings. Realist evaluation is a methodology that explicitly recognizes this. It involves constructing, testing, and refining stakeholders’ ideas or theories about how and in what contexts a technology is supposed to work. These theories detail how particular contexts shape users’ responses to components of the technology (intervention mechanisms) to generate outcomes. They are presented as context-mechanism-outcome (CMO) configurations, where context (C) + mechanism (M) = outcome (O). In this way, and in comparison to more general qualitative approaches, realist evaluation moves beyond listing barriers and facilitators, offering specificity in understanding the relationship between contexts, mechanisms, and outcomes.

The elicitation of stakeholders’ theories can be done in a number of ways, such as interviewing stakeholders, reviewing the existing literature on the topic, identifying relevant theories from the sociological or other works of literature, or some combination of these approaches. To ensure we are building on existing work, we have chosen to elicit stakeholders’ theories through a review of the literature related to the use of AI in pathology. In contrast to a full realist review, where published evidence is used to test and refine stakeholders’ theories [19], theories elicited in this review will be refined through interviews with pathologists in later stages of the research.

While this is not a full realist review, where relevant, we have reported the items included in the Realist and Meta-narrative Evidence Synthesis: Evolving Standards (RAMESES) reporting guidelines for the reporting of realist reviews [20] (see Multimedia Appendix 1 for completed RAMESES checklist).

### Search Strategy

The overriding question for the review was what works, for whom, in what circumstances, and how to encourage uptake and impact of AI in pathology. To address this question, several searches were undertaken. Search 1 sought studies, reports, and policy documents from the following databases: arXiv.org (Cornell University) repository, Ovid MEDLINE(R), and HMIC Health Management Information Consortium (Ovid). Searches were developed for the concepts of artificial intelligence and histopathology. Subject headings and free text words were identified for use in the search concepts by an information specialist (JW) and project team members. Further terms were identified and tested from known relevant papers. Search results were limited to English-language publications. We also limited search results to publications published since 2000, given the recent development of AI in pathology. The searches were peer-reviewed by a second information specialist using the Peer Review of Electronic Search Strategies (PRESS) checklist (see Multimedia Appendix 2 for full search strategies) [21].

Search 2 sought reports discussing AI, authored by Eric Topol in Ovid MEDLINE(R), Sciences Citation Index (Clarivate Analytics Web of Science), and Emerging Sources Citation Index (Clarivate Analytics Web of Science; see Multimedia Appendix 3). While searching for a particular author is not typically used in traditional systematic reviews, named author searches are recognized as a method for increasing the number of outputs included in a review [22]. Such an approach is recommended for realist reviews [23] and more generally for understanding the theories that underpin complex interventions and the contexts in which they are implemented [22]. Named author searches are used in the theory elicitation stage of realist reviews as a means of gathering opinion pieces, remembering that we are looking for theories rather than empirical evidence [24]. Eric Topol was chosen as an “opinion leader,” given his popular science book on the topic of AI in health care [25] and his input into England’s strategy on digital health training and education, including around AI [26].

Searches were also undertaken of the following websites: the Food and Drugs Administration, the College of American Pathologists, the Royal College of Pathologists, and the Digital Pathology Association along with a number of Google searches (see Multimedia Appendix 4). Additional papers were identified through personal recommendation and “snowballing” (pursuing references of references) [27]. Search results were collated and deduplicated in EndNote (Clarivate).

### Selection and Appraisal of Documents

As the aim of this review is to identify and characterize stakeholders’ perspectives regarding contextual factors that will enhance or restrict the uptake of AI in pathology rather than to assess the validity of these perspectives, the identified papers were screened not based on rigor but on relevance to the review question. Titles and abstracts returned from the search results were screened in EndNote, asking the following questions: is the paper about AI in pathology or health care? And does the paper contain ideas about how, for whom, and in what circumstance AI can work (in the clinical setting)? Full-text copies of potentially relevant documents were then obtained and read by the reviewers to identify if they contained ideas about the introduction of AI into pathology or other relevant health care settings.

While these searches were primarily concerned with pathology, papers from outside this field (particularly radiology) were also returned. This is due to the medical use of “pathology” to describe the features typical of the way a disease presents, in addition to it being the branch of medicine that deals with the analysis of body tissue for diagnostic or forensic purposes. These tangential papers were retained as they potentially contained concepts that were transferable or relevant to the field of pathology. Similarly, a number of papers concerned with image analysis without the use of AI were retained on the basis that some of the potential supports and constraints to implementation they described would have relevance to the use of AI-based image analysis.

### Data Extraction, Analysis, and Synthesis

Documents were entered into NVivo 12 (QSR International Pty Ltd) software for qualitative analysis. Sections of text were indexed in an iterative process using a series of codes that evolved to represent topics relevant to the review question. Following the realist strategy, these codes sought to capture different contexts, mechanisms, and outcomes that could affect the introduction of AI in pathology. The coded data were used to produce narrative summaries of each of the identified contexts, mechanisms, and outcomes. The reviewers and other authors then discussed these narratives and translated them into CMO configurations.

While the documents provided data about outcomes associated with the use of AI and some data could be drawn out about contexts, there was little about the mechanisms through which these were achieved. Therefore, to guide our thinking, we also drew on substantive theories concerning the implementation of technology and complex interventions more generally, identifying potentially relevant theories through team discussions of the emerging themes. Use of substantive theory is in line with the realist approach, which argues that the design of interventions tends to be based on a limited number of theories regarding human behavior and therefore, rather than starting from scratch when evaluating a new intervention, researchers should also make use of existing theory [16].

## Results

### Overview

The search identified 1420 papers, 4 web pages, and 9 government or institution or foundation documents, providing a total of 1433 unique records (see Multimedia Appendix 5 for PRISMA [Preferred Reporting Items for Systematic Reviews and Meta-Analyses] diagram). After title and abstract screening, 1294 documents were determined to not be relevant, leaving 139 potentially relevant documents. After screening of full texts of these, 101 documents were identified as relevant. All 101 documents were coded, although there was much repetition of the themes contained within them. Below we summarize these themes, organizing them according to the realist concepts of context, mechanism, and outcome. As realist evaluation typically starts with looking at outcome patterns before identifying the contexts and mechanisms that lead to the outcome pattern, the anticipated impacts (outcomes), both positive and negative, of AI in pathology were first considered. The contexts that may impact uptake and impacts of AI were then considered. The mechanisms that may be triggered in particular contexts for the outcomes to be achieved were finally considered. The analysis also suggested practical challenges of introducing AI, such as infrastructure and the need for adequate training data, but we do not discuss these in what follows as our focus is on contextual factors that are likely to shape pathologists’ responses to AI.

### Impacts of AI in Pathology

#### Accuracy

The most commonly mentioned benefit of the use of AI, whether anticipated or determined experimentally, was increased accuracy [8,28-31]. Some authors have argued that currently pathology is a subjective specialty relying on manual observation subject to human skill, procedural errors, or inefﬁciencies in processes and bias [32]. From this perspective, increased accuracy results from the standardization of diagnosis through quantitative evaluation of samples [7,29,32-37]. Increased accuracy was also seen as a consequence of using AI to remove variance in results attributed to decrease in pathologist performance that occurs when working under time constraints [38]. It has also been argued that, if AI is used to augment the decision-making of pathologists, it may increase the knowledge of pathologists, and in that way increase accuracy both when AI is used and when the pathologist works alone [39].

#### Speed

Another predicted benefit of AI in pathology was the ability of computers to make a diagnosis quickly. This was expressed as increasing the speed of the diagnosis [28,40,41], improving workflow [29,42], keeping pace with increasing demand [28,43], and increasing efficiency [26,44]. One way in which authors theorized this increased efficiency could be achieved was by reducing the number of slides that the pathologist has to look at [34]. For example, in a screening, triage, or prioritization scenario, the pathologist would be presented with all positive slides for rapid review, while in a diagnostic or fully automated scenario, the pathologist would not need to review all benign slides, as the AI tool would review them instead. This could remove a significant percentage of slides from the workload of a pathologist. Another way in which it was theorized this could be achieved was through identifying regions of interest in a slide [45]. An anticipated knock-on benefit of increasing efficiency was addressing the shortage of pathologists [29].

#### Combination of Data Sources

The ability to analyze multiple disparate data sources was highlighted as another possible benefit of AI. This would include combining images with patient records [8], combining pathology with other imaging techniques (magnetic resonance imaging, computerized tomography, and x-ray) [6,46], and integrating pathology with genomics, metabolomics, and other diagnostic techniques (eg, Raman spectroscopy) [32,42,46]. While currently hypothetical, authors argue that this has the possibility to better characterize disease [6,47], which may lead to new or enhanced predictive models [48].

#### Role of the Pathologist

Another potential impact is on the role of the pathologist. Most authors opine that pathologists are unlikely to be replaced by AI [3,28,33,49] but that their role is likely to change substantially [8,49,50], echoing what has regularly happened when disruptive technology has been introduced [51]. In the immediate term, it is thought that the role of the pathologist is unlikely to change, as currently, the field of AI in image analysis is in its infancy. Some have argued that, if anything, the pathologist will be more indispensable than ever since their knowledge will be essential for algorithm design [3,8,35] and the generation of annotated data for AI training [33]. Furthermore, there will always be a role in assessing whether enough tissue of sufficient quality (eg, lacking artifacts and representative of the targeted lesion) is present at the tissue processing stage and directing an AI algorithm to assess specific areas of tissue identified as of interest by a pathologist. Jha and Topol [52] and others [53] theorize an alternative view of the future where, as AI for image analysis improves, pathologists, along with radiologists, will become information specialists, managing information extracted by AI in the clinical context of the patient rather than extracting information from images themselves.

### Contexts for AI Use

#### Collaborative Working

In the literature, we identified 2 key contextual factors that authors argue have the potential to affect whether or not the anticipated benefits of AI are achieved. The first of these is the size of and expertise within a department and to what extent the pathologists within that department work collaboratively. Studies looking at the accuracy of AI have typically compared AI with the decision-making of a single pathologist, and many papers present a scenario of the pathologist working alone (with or without the support of AI). However, Campanella et al [54] note the collaborative nature of pathology, arguing that, with access to additional information provided by immunohistochemistry, it could be assumed “that a team of pathologists at a comprehensive cancer centre will operate with 100% sensitivity and specificity,” and therefore, AI in such a context should not seek to achieve the impossible goal of surpassing the performance of pathologists. Campanella et al [54] go on to argue that, in such a context, the focus should be on the AI achieving 100% sensitivity with an acceptable false positive rate, so that pathologists can focus on those cases and slides where the AI has identified a tumor, thereby increasing efficiency. While some may question this claim of 100% sensitivity and specificity, the underlying theory seems to be that, when pathologists work collaboratively to generate consensus diagnoses, sensitivity and specificity are likely to be greater than when a pathologist is working alone. Conversely, a theory stemming from this is that AI could provide greater benefit in terms of increasing accuracy in smaller, nonspecialist departments. Thus, while AI is often touted as a replacement for a variable or inconsistent human opinion, in the real world, pathologists can get opinions from others and use ancillary testing to confirm diagnoses. So, depending on the clinical context, accuracy at a single point in the diagnostic process may be less important than the overall output of the process.

#### Regulation

The second contextual factor is that of regulation. A key issue raised is the tension between rapid technological advancement and safety [55]. Allen [28] describes the difficulty of balancing the need to ensure patient safety without stifling development. A particular issue is the lack of transparency about how AI algorithms work; it is not possible to see inside the “black box” of their decision-making and know what features the algorithm is using to make its decision [8,37,39,40,56]. Some have argued that one of the benefits of AI is the potential to reduce medical malpractice liability by improving diagnosis and treatment, reducing medical error, and preventing ineffective and unnecessary care [28]. However, concerns regarding how the use of AI will impact pathologists’ liability are more dominant in the literature, suggesting that a failure to resolve these issues could constrain the uptake of AI. Some have argued that the rise of AI in health care challenges the traditional liability structures used if AI moves beyond augmenting the work of pathologists and begins to, for example, automate certain tasks [28,57]. A survey of pathologists found that almost 50% thought that the platform vendor should bear some of the liability [58].

### Mechanisms

In realist evaluation, mechanisms are understood as a combination of the resources that a technology provides and the users’ responses to those resources. The literature described a broad range of actual and potential technologies that could be considered as resources. These different technologies can be conceptualized at a more abstract level, using a typology that we identified in the literature. This typology describes 3 different models of the relationship between the pathologist and AI [28].

In the first model, the pathologist is “in the loop,” or what some refer to as the “augmented pathologist” [56], where AI is a tool that pathologists use to aid their diagnosis [3,31,33,35]. It has been proposed that this is the model that we are most likely to see in the short term. Some have suggested that AI should be seen as a colleague who can provide second opinions on difficult cases, but without individual challenges, such as tiredness, and collaborative challenges, such as those that result from hierarchies [36]. The AI-human combination is believed to be more accurate because the errors made by AI are not strongly correlated with the errors made by humans [39].

In the second model, the pathologist will become “on the loop,” and some authors suggest we will see this in the medium term [28]. In this scenario, AI is capable of making independent decisions but pathologists are still involved. This could initially be a system whereby benign or normal tissue is screened out at an early stage leaving the pathologist to concentrate on diseased tissue. As algorithms further advance, it is possible that AI diagnoses more and more conditions, with the pathologist only needed for highly unusual or ambiguous cases. In this situation, the pathologist plays a role in quality control and oversight, checking that the decisions being made are appropriate rather than making the decisions.

In the third model, the pathologist is “out of the loop,” a scenario that some authors predict we may reach in the long term [28], at least for some decisions. It has been suggested AI could automate routine tasks [59] and more time-consuming tasks [32,60]. It is theorized that this will enable pathologists to spend more time on high-level decision-making tasks, particularly those related to disease presentations with more confounding features [60]. Tasks that have been identified in the literature for automation include those “tedious routine diagnostic tasks that require great accuracy” such as finding metastases in lymph node sections, with the potential for a significant reduction in the workload of pathologists [38]. In this situation, AI has become autonomous, and decision-making has shifted away from human control. This may be unlikely to happen in the foreseeable future, and the general consensus in the literature is that there will always be human involvement in the diagnostic process [26,28,36,37,46,53,55-57,61-65].

How pathologists might reason about and respond to these different models of working alongside AI was less clear from the literature. However, broader literature on the use of AI in health care provides some useful insights about which of these models is most likely to be responded to positively. For uptake of AI, the literature suggests that there is the need for advice in a way that recognizes the expertise of the user, making it clear that it is designed to inform and assist but not replace the clinician [66], implying that the scenario of the pathologist being “in the loop” will be most acceptable to pathologists. In thinking about this scenario, we drew on substantive theories regarding the implementation of technology and complex interventions more generally. Here, normalization process theory (NPT) provided valuable understanding. Successful introduction of technology involves interactions between individual clinicians and their work environment until the technology becomes embedded (routinely incorporated into everyday work) and integrated (sustained over time) into routine practice, a process known as “normalization” [67]. NPT suggests that, for normalization to occur, 4 key constructs need to be considered: coherence: sense-making—where individuals make sense of the new technology and how it differs from existing practice; cognitive participation: the process of engaging individuals with the introduction of the technology; collective action: how the work processes are adapted and altered to make the intervention happen; and reflexive monitoring: the formal and informal appraisal of the benefits and costs of the intervention [67-69]. This suggests that if pathologists have been able to “make sense” of AI, have been engaged in the adoption process, have been able to adapt their work processes, and are able to identify potential benefits to its introduction, it is more likely to become embedded into practice.

We also looked at theories relating to the adoption of a clinical decision support system (CDSS), with AI being a form of CDSS [66]. Relevant theories are the user acceptance and system adaptation design model and the input-process-output-engage (IPOE) model [70]. The user acceptance and system adaptation design model suggests that, for users to accept a CDSS, an iterative design process with early end-user involvement is needed, along with rigorous usability testing in both laboratory and natural settings, to ensure that the system works within the cognitive and environmental constraints of the intended user. The IPOE model suggests that acceptance of CDSS requires the CDSS to provide users with the rules that the machine followed to generate the output, so the user can make informed decisions when deciding whether to follow the recommendation.

A challenge, highlighted by NPT and the IPOE model, may be making sense of the black box of AI. In the pathology literature, it is theorized that trust is needed for uptake of AI [71]. Drawing on studies of AI implementation in other health care settings, we can also theorize that pathologists’ trust will be eroded when the AI recommendations conflict with their own observations and experience [12]. Consequently, it is theorized that the “resource” of explainable AI is necessary for building pathologists’ trust and will increase acceptance of the scenario of pathologists being “on the loop” [71].

## Discussion

### Principal Findings

This review has described the anticipated benefits of AI in digital pathology. While these benefits are not surprising, reflecting broader claims made for AI in the health care literature—namely, increased accuracy and efficiency—the value of this review comes from the use of a realist approach, which has allowed us to go beyond a simple listing of benefits to theorizing the contexts in which, and the mechanisms through which, such benefits are likely to be achieved. These are summarized as CMO configurations in Table 1. It has also enabled us to draw on a wider range of literature than in more traditional review approaches, an important feature given the absence of studies exploring pathologists’ attitudes toward AI [14].

**Table 1:** The “context + mechanism = outcome” configurations.

Context	Mechanism		Outcome
	Resource	Response	
Iterative design process with early user involvement Pathologists involved in the decision to implement AI^a^ in their department and are given the opportunity to regularly feed into reviews of its benefits and costs	AI undertakes quantitative tasks High usability Integration into workflow	The pathologist is confident in ability of AI to undertake quantitative tasks and so is willing to trust the output and incorporate it into their decision-making	Increased efficiency Increased accuracy
Iterative design process with early user involvement Pathologists involved in the decision to implement AI in their department and are given the opportunity to regularly feed into reviews of its benefits and costs	AI identifies regions of interest Explainable AI High usability Integration into workflow	Understanding the basis on which regions of interest have been identified, the pathologist is confident that all relevant regions of interest have been identified, reducing the percentage of slides, and the amount of slides they need to look at	Increased efficiency
Iterative design process with early user involvement Pathologists involved in the decision to implement AI in their department and are given the opportunity to regularly feed into reviews of its benefits and costs Smaller, nonspecialist departments	Provision of “second opinion” Explainable AI High usability Integration into workflow	Understanding the basis on which the opinion is made, the pathologist is willing to trust and accept the opinion	Increased accuracy
Iterative design process with early user involvement Pathologists involved in the decision to implement AI in their department and are given the opportunity to regularly feed into reviews of its benefits and costs Specialized team	AI screens out negative cases Explainable AI High usability Integration into workflow	Understanding the basis on which positive cases have been identified, the team is confident that all positive cases have been identified, reducing the number of cases they need to look at	Reduced workload

^a^AI: artificial intelligence.

We began this review with the intention of identifying stakeholders’ theories about the contextual factors that may support or constrain the adoption of AI in pathology. However, the review revealed a gap in the literature regarding the discussion of this topic, likely a reflection of the current state of progress in the development of AI in pathology. As we begin to introduce AI into pathology, there is a need for empirical research to address this gap. However, this points to an additional benefit of a realist approach, which allowed us to integrate existing theory concerning the implementation of technology and complex interventions more generally. This enabled us to develop some tentative theories regarding the mechanisms that may lead pathologists to choose to integrate AI into their work practice, providing a strong theoretical basis for future research in this area.

The review has several implications for the design and reporting of studies as we begin to move from experimental studies to real-world evaluation and the use of AI in pathology departments. Firstly, the findings highlight tasks where AI is likely to provide the greatest benefit and which benefits are most desirable in a given setting. For example, in large specialist centers, the emphasis should be on reducing workload rather than increasing accuracy. This responds to calls for developers to carefully consider the tasks that are best performed by AI and those best performed by clinicians [12]. Alternatively, the review findings can inform the selection of sites for evaluation. For example, if the ambition is to increase the accuracy of diagnosis, then smaller, nonspecialist departments are a more appropriate choice than large specialist centers. Designers also need to give careful thought to usability and how AI is integrated into the pathologist’s workflow.

The findings and resulting CMO configurations also suggest that, except for simple quantitative tasks, explainable AI will be needed for pathologists to trust the recommendations provided. However, this may be based on a misunderstanding of the current nature of explainable AI; broad descriptions of how the AI system works in a general sense can be produced but they are rarely informative with respect to individual decisions [72]. For example, in radiology, the trustworthiness of saliency maps, a widely used method to provide explainable AI in medical imaging, has been questioned [73]. Instead, rigorous evaluations should be used to provide evidence of the trustworthiness of AI, as is the case with other black-box systems in health care, such as medicines where the mechanisms of action are only partially understood [72].

Following on from this, the review findings highlight information that should be reported in evaluation studies of AI, regarding not only the size and nature of the department but also how pathologists worked in the different departments, working alone or in teams. The issue of reporting AI studies in health care has been highlighted by other authors, with recommendations for describing the study setting, the target user, the digitized workflow, and the extent of use [74-78]. Our analysis suggests that for real-world studies, a finer-grained level of reporting would be beneficial; by capturing and reporting such details, it will enable identification of the specific contexts in which AI is likely to provide the greatest benefit and therefore, where its implementation is most justified. To capture this information regarding the extent of collaborative work, as well as to capture information about the contextual factors that support or constrain the use of AI in different departments, real-world trials need to be complemented by mixed method or qualitative process evaluations.

The findings also have implications for the introduction of AI into pathology. Pathologists should be involved in the decision to introduce AI and have the opportunity to feed into evaluations of the costs and benefits of the system following its introduction.

### Limitations

The CMO configurations presented earlier are tentative theories, as they have not been tested with empirical data. In the next stage of this research, these will be explored and refined through interviews with pathologists. This will provide the basis for a future realist evaluation of AI in pathology, gathering empirical data to test the theories.

### Conclusions

This paper has presented a review of stakeholders’ theories of how and in what contexts AI will be adopted and provide benefit within pathology. The results suggest that for uptake of AI in pathology, for all but the simplest quantitative tasks, measures will be required that either increase confidence in the system or provide users with an understanding of the performance of the system. For specialist centers, efforts should focus on reducing workload, rather than increasing accuracy. Designers also need to give careful thought to usability and how AI is integrated into pathologists’ workflow.

## Appendix

Multimedia Appendix 1 Realist and Meta-narrative Evidence Synthesis: Evolving Standards (RAMESES) checklist.

Multimedia Appendix 2 Database searches.

Multimedia Appendix 3 Author search.

Multimedia Appendix 4 Web searches.

Multimedia Appendix 5 PRISMA (Preferred Reporting Items for Systematic Reviews and Meta-Analyses) diagram.

## Abbreviations

AI: artificial intelligence
CDSS: clinical decision support system
CMO: context-mechanism-outcome
IPOE: input-process-output-engage
NPT: normalization process theory
PRESS: Peer Review of Electronic Search Strategies
PRISMA: Preferred Reporting Items for Systematic Reviews and Meta-Analyses
RAMESES: Realist and Meta-narrative Evidence Synthesis: Evolving Standards
WSI: whole slide imaging
