# Supplementary material for: What Works Where and How for Uptake and Impact of Artificial Intelligence in Pathology: Review of Theories for a Realist Evaluation
Source: J Med Internet Res. 2023 Apr 24;25:e38039. doi: 10.2196/38039 (PMC10167589; doi:10.2196/38039)
Supplement: Multimedia Appendix 2 [file jmir_v25i1e38039_app2.docx]

**What works where and how for uptake and impact of artificial intelligence in pathology: A review of theories for a realist evaluation (King et al.)**

**Multimedia Appendix 2. Database searches.**

***arXiv.org (Cornell University)***

date_range: from 2000-01-01 to 2019-08-14;

include_cross_list:

title=Pathology OR Pathologist;

AND

all="artificial intelligence" OR "machine learning" OR "deep learning" OR "image analysis"

***HMIC Health Management Information Consortium (Ovid) 1983 – present***

1 "artificial* intelligen*".ti,ab,hw. (65)

2 AI.ti,ab. (50)

3 image? analyz*.ti,ab,hw. (0)

4 image? analys*.ti,ab,hw. (8)

5 machine learning.ti,ab,hw. (25)

6 deep learning.ti,ab,hw. (11)

7 artificial intelligence/ or expert systems/ (151)

8 expert system*.ti,ab,hw. (171)

9 or/1-8 [AI] (294)

10 pathology.ti,ab,hw. (1260)

11 histopathology.ti,ab,hw. (90)

12 exp pathology/ (320)

13 exp pathologists/ (62)

14 (pathologist* or histopathologist* or neuropathologist*).ti,ab,hw. (196)

15 or/10-14 [Pathology] (1410)

16 9 and 15 (4)

17 limit 16 to yr="2000 -Current" (1)

***Ovid MEDLINE(R) 1946 to August week 2 2019 and Epub Ahead of Print, In-Process & Other Non-Indexed Citations and Daily < August 12, 2019>***

1 artificial intelligence/ or machine learning/ or deep learning/ or supervised machine learning/ or support vector machine/ or unsupervised machine learning/ (34043)

2 "artificial* intelligen*".ti,ab,kw. (5179)

3 AI.ti,ab,kw. (23715)

4 (image? analyz* and (neural network* or algorithm* or generative adversarial network*)).ti,ab,kw. (48)

5 (image? analys* and (neural network* or algorithm* or generative adversarial network*)).ti,ab,kw. (3240)

6 machine learning.ti,ab,kw. (21817)

7 deep learning.ti,ab,kw. (5244)

8 expert system*.ti,ab,kw. (2932)

9 or/1-8 [AI] (81181)

10 pathology.ti,ab,kw. (291457)

11 histopathology.ti,ab,kw. (59303)

12 pathology/ or forensic pathology/ or neuropathology/ or pathology, clinical/ or pathology, molecular/ or pathology, surgical/ or telepathology/ (46447)

13 Pathologists/ (326)

14 (pathologist* or histopathologist* or neuropathologist*).ti,ab,kw. (35146)

15 or/10-14 [Pathology] (384444)

16 9 and 15 (1682)

17 exp animals/ not humans/ (4607932)

18 16 not 17 (1574)

19 limit 18 to yr="2000 -Current" (1394)

20 limit 19 to english language (1354)
