# Supplementary material for: What Works Where and How for Uptake and Impact of Artificial Intelligence in Pathology: Review of Theories for a Realist Evaluation
Source: J Med Internet Res. 2023 Apr 24;25:e38039. doi: 10.2196/38039 (PMC10167589; doi:10.2196/38039)
Supplement: Multimedia Appendix 3 [file jmir_v25i1e38039_app3.docx]

**What works where and how for uptake and impact of artificial intelligence in pathology: A review of theories for a realist evaluation (King et al.)**

**Multimedia Appendix 3. Author search.**

***Ovid MEDLINE(R) 1946 to August week 2 2019 and Epub Ahead of Print, In-Process & Other Non-Indexed Citations and Daily August 16, 2019***

1 artificial intelligence/ or machine learning/ or deep learning/ or supervised machine learning/ or support vector machine/ or unsupervised machine learning/ (34132)

2 "artificial* intelligen*".ti,ab,kw. (5211)

3 AI.ti,ab,kw. (23730)

4 (image? analyz* and (neural network* or algorithm* or generative adversarial network*)).ti,ab,kw. (48)

5 (image? analys* and (neural network* or algorithm* or generative adversarial network*)).ti,ab,kw. (3240)

6 machine learning.ti,ab,kw. (21919)

7 deep learning.ti,ab,kw. (5299)

8 expert system*.ti,ab,kw. (2934)

9 or/1-8 [AI] (81363)

10 exp animals/ not humans/ (4609651)

11 topol eric*.au. (0)

12 topol e.au. (51)

13 topol ej*.au. (1125)

14 or/11-13 (1176)

15 9 and 14 (8)

***Sciences Citation Index (Clarivate Analytics Web of Science) 1900-present and Emerging Sources Citation Index (Clarivate Analytics Web of Science) 2015-present***

# 3 11 #2 AND #1

# 2 200,106 TOPIC: ("artificial* intelligen*".) OR TOPIC: ("image* analyz*" and (neural network* or algorithm* or generative adversarial network*)) OR TOPIC: ("image* analys*" and (neural network* or algorithm* or "generative adversarial network*")) OR TOPIC:(machine learning) OR TOPIC: (deep learning) OR TOPIC: (expert system) OR TOPIC: (AI)

# 1 1,817 AUTHOR: (topol eric*) OR AUTHOR: (topol e) OR AUTHOR: (topol e.j.)
