# Supplementary material for: What Works Where and How for Uptake and Impact of Artificial Intelligence in Pathology: Review of Theories for a Realist Evaluation
Source: J Med Internet Res. 2023 Apr 24;25:e38039. doi: 10.2196/38039 (PMC10167589; doi:10.2196/38039)
Supplement: Multimedia Appendix 4 [file jmir_v25i1e38039_app4.docx]

**What works where and how for uptake and impact of artificial intelligence in pathology: A review of theories for a realist evaluation (King et al.)**

**Multimedia Appendix 4. Web searches.**

| **Website and URL** | **Date searched** | **Search strategy** | **Number of records found** | **Number records looked at on screen** | **Notes – did you browse? Use advanced search interface?** |
| --- | --- | --- | --- | --- | --- |
| FDA  <https://www.fda.gov/>  <https://www.fda.gov/about-fda/about-website/fdagov-archive> | 19/09/19 | Used search feature for: artificial intelligence  Used search feature for: artificial intelligence | 258  77 | 60, no new documents found  77, downloaded 1 | Used advanced search and limited to PDF format |
| College of American Pathologists  <https://www.cap.org/> | 27/09/19 | Used search feature for: artificial intelligence | 83 | 83, no relevant documents found. | Also browsed, nothing else found |
| Royal College of Pathologists  <https://www.rcpath.org/> | 27/09/19 | Used search feature for: artificial intelligence | 24 | 24. Saved two. One blog post and the response to the Topol Review |  |
| Digital Pathology Association <https://digitalpathologyassociation.org/> | 27/09/19 | Used search feature for: artificial intelligence | 40 | 40. None downloaded, all related to talks given/conferences |  |
| Digital Pathology Association <https://digitalpathologyassociation.org/> | 27/09/19 | Browsing | 20 min | 3 white papers, 2 relevant but already found by previous searches  3 blog posts relevant |  |
| Google  <https://www.google.com/advanced_search> | 19/09/19  Search 1 | See search terms below | 130000 | Too many articles already returned by literature searches. Needed alternative terms | Used advance search, see below |
|  | 19/09/19  Search 2 | See search terms below | 2040 | 45. Two selected | Used advance search |
|  | 19/09/19  Search 3 | See search terms below | 165 | 45. none selected | Used advance search |
|  | 19/09/19  Search 4 | See search terms below | 554 | 45. 3 selected | Used advance search |
|  | 20/09/19  Search 5 | See search terms below | 9983 | 45. no new articles | Used advance search |
|  | 20/09/19  Search 6 | See search terms below | 1350 | 45. 2 selected | Used advance search |
|  | 20/09/19  Search 7 | See search terms below | 1600 | 45. no new articles | Used advance search |

**Google search terms**

Search 1

| **Find pages with** | |
| --- | --- |
| All these words | Digital pathology |
| This exact word or phrase | Artificial intelligence |
| Any of these words |  |
| None of these words |  |
| Number ranging from |  |
| **Then narrow results by** | |
| Language | Any language |
| Region | Any region |
| Last updated | Anytime |
| Site or domain |  |
| Terms appearing | Anywhere in the page |
| SafeSearch | Show most relevant first |
| File type | Adobe Acrobat PDF (.pdf) |
| Usage rights | Not filtered by licence |

Search 2

| **Find pages with** | |
| --- | --- |
| All these words | Digital pathology |
| This exact word or phrase | Artificial intelligence |
| Any of these words |  |
| None of these words |  |
| Number ranging from |  |
| **Then narrow results by** | |
| Language | Any language |
| Region | Any region |
| Last updated | Anytime |
| Site or domain | .gov |
| Terms appearing | Anywhere in the page |
| SafeSearch | Show most relevant first |
| File type | Adobe Acrobat PDF (.pdf) |
| Usage rights | Not filtered by licence |

Search 3

| **Find pages with** | |
| --- | --- |
| All these words | Digital pathology |
| This exact word or phrase | Artificial intelligence |
| Any of these words |  |
| None of these words |  |
| Number ranging from |  |
| **Then narrow results by** | |
| Language | Any language |
| Region | Any region |
| Last updated | Anytime |
| Site or domain | .gov.uk |
| Terms appearing | Anywhere in the page |
| SafeSearch | Show most relevant first |
| File type | Adobe Acrobat PDF (.pdf) |
| Usage rights | Not filtered by licence |

Search 4

| **Find pages with** | |
| --- | --- |
| All these words | Digital pathology |
| This exact word or phrase | Artificial intelligence |
| Any of these words |  |
| None of these words |  |
| Number ranging from |  |
| **Then narrow results by** | |
| Language | Any language |
| Region | Any region |
| Last updated | Anytime |
| Site or domain | .NHS.uk |
| Terms appearing | Anywhere in the page |
| SafeSearch | Show most relevant first |
| File type | Adobe Acrobat PDF (.pdf) |
| Usage rights | Not filtered by licence |

Search 5

| **Find pages with** | |
| --- | --- |
| All these words | Artificial intelligence |
| This exact word or phrase | Pathology |
| Any of these words |  |
| None of these words |  |
| Number ranging from |  |
| **Then narrow results by** | |
| Language | Any language |
| Region | Any region |
| Last updated | Anytime |
| Site or domain | .gov |
| Terms appearing | Anywhere in the page |
| SafeSearch | Show most relevant first |
| File type | Adobe Acrobat PDF (.pdf) |
| Usage rights | Not filtered by licence |

Search 6

| **Find pages with** | |
| --- | --- |
| All these words | Artificial intelligence |
| This exact word or phrase | Pathology |
| Any of these words |  |
| None of these words |  |
| Number ranging from |  |
| **Then narrow results by** | |
| Language | Any language |
| Region | Any region |
| Last updated | Anytime |
| Site or domain | .gov.uk |
| Terms appearing | Anywhere in the page |
| SafeSearch | Show most relevant first |
| File type | Adobe Acrobat PDF (.pdf) |
| Usage rights | Not filtered by licence |

Search 7

| **Find pages with** | |
| --- | --- |
| All these words | Artificial intelligence |
| This exact word or phrase | Pathology |
| Any of these words |  |
| None of these words |  |
| Number ranging from |  |
| **Then narrow results by** | |
| Language | Any language |
| Region | Any region |
| Last updated | Anytime |
| Site or domain | .NHS.uk |
| Terms appearing | Anywhere in the page |
| SafeSearch | Show most relevant first |
| File type | Adobe Acrobat PDF (.pdf) |
| Usage rights | Not filtered by licence |
