# Supplementary material for: What Works Where and How for Uptake and Impact of Artificial Intelligence in Pathology: Review of Theories for a Realist Evaluation
Source: J Med Internet Res. 2023 Apr 24;25:e38039. doi: 10.2196/38039 (PMC10167589; doi:10.2196/38039)
Supplement: Multimedia Appendix 5 [file jmir_v25i1e38039_app5.docx]

**What works where and how for uptake and impact of artificial intelligence in pathology: A review of theories for a realist evaluation (King et al.)**

**Multimedia Appendix 5.** Preferred Reporting Items for Systematic Reviews and Meta-Analyses **(PRISMA) diagram.**

Records removed *before screening*:

Duplicate records removed (n = 82)

Records identified from:

Databases (n = 1497)

Other sources (n = 18)

**Identification**

Records screened

(n = 1433)

Records excluded

(n = 1294)

Reports sought for retrieval

(n = 139)

Reports not retrieved

(n = 0)

**Screening**

Reports assessed for eligibility

(n = 139)

Reports excluded

(n = 38)

Reports included in review

(n = 101)

**Included**
